# Role of LsrR in the regulation of biofilm formation in mammary pathogenic *Escherichia coli*

**DOI:** 10.1186/s12917-024-04086-9

**Published:** 2024-05-23

**Authors:** Li Xu, Wei Wang, Xin Zhang, Kai Ma, Hui Wang, Ting Xue

**Affiliations:** School of Life Sciences, Anhui Agricultural University, Hefei,, Anhui 230036 China

**Keywords:** MPEC, Mastitis, LsrR, Biofilm

## Abstract

**Background:**

Mammary Pathogenic *Escherichia coli* (MPEC) is an important pathogen that can escape the attack of the host immune system through biofilm formation and proliferate in the mammary gland continuously, resulting in mastitis in cows and causing enormous economic losses. As an effector of AI-2 quorum sensing, LsrR extensively affects the expression levels of hundreds of genes related to multiple biological processes in model *E. coli* strain. However, the regulatory role of LsrR in MPEC and whether it is involved in pathogenesis has been seldom reported.

**Results:**

In this study, the function of LsrR in strain MPEC5, obtained from a milk sample in dairy cows with mastitis, was investigated by performing high-throughput sequencing (RNA-seq) assays. The results revealed that LsrR down-regulated the transcript levels of *fimAICDFGH* (encoding Type 1 pili), which have been reported to be associated with biofilm formation process. Biofilm assays confirmed that deletion of *lsrR* resulted in a significant increase in biofilm formation *in vitro.* In addition, electrophoretic mobility shift assay (EMSA) provided evidence that LsrR protein could directly bind to the promoter regions of *fimAICDFGH* in a dose-dependent manner.

**Conclusions:**

These results indicate that LsrR protein inhibits the biofilm formation ability of MPEC5 by directly binding to the *fimAICDFGH* promoter region. This study presents a novel clue for further exploration of the prevention and treatment of MPEC.

**Supplementary Information:**

The online version contains supplementary material available at 10.1186/s12917-024-04086-9.

## Introduction

*Escherichia coli* (*E. coli*) is one of the main causative agents of clinical mammary mastitis [1–3], which results in enormous economic losses to the dairy industry worldwide. Mammary pathogenic *Escherichia coli* (MPEC) represents a subset of extraintestinal pathogenic *E. coli* strains [4]. These bacteria exhibit an affinity for adhering to and infecting epithelial cells within the mammary glands of dairy cows alongside other pathogens, culminating in the eventual formation of biofilms [5]. Biofilm is a complex polysaccharides-protein complex composed of bacteria attached to living or non-living surfaces and extracellular polymers (EPS) secreted by bacteria [6, 7]. Pathogens can evade the immune system and multiply constantly in the mammary gland of the host by forming biofilm, causing intramammary infections persistently [8, 9]. Thus, it becomes important to study the role of biofilms in the pathogenesis of mastitis.

Type 1 fimbriae, one of the important virulence factors and the most common adhesive organelles in the members of the *Enterobacteriaceae* family [10] are mainly responsible for the initial contact with host cells and for the interactions of host-pathogen [11]. The *fim* gene cluster (*fimA ~ H*) encodes Type 1 fimbrial proteins [12], where FimA (encoded by *fimA*) serves as the primary structural subunit. Additionally, the structural subunit FimH (encoded by *fimH*) exhibits the ability to facilitate bacterial adhesion by engaging the mannose-containing glycoprotein receptors present in host cells. Previous research has suggested the important role of type 1 fimbriae in the initial phase and maturation stage of biofilm formation [10, 13, 14], but the detailed molecular mechanism is unclear, and needs to be further studied.

Autoinducer 2 (AI-2), produced by LuxS in many species of Gram-negative and Gram-positive bacteria, is proposed to be a quorum sensing (QS) signaling molecule related to interspecific communication [15, 16]. The extracellular AI-2 is imported into the cells of *E. coli* by an ATP-binding transporter, encoded by the *lsrACDB* operon. The expression of *lsr* operon is regulated by LsrK, a cognate signal kinase, and LsrR, a DNA-binding repressor. Both of *lsrR* and *lsrK* genes are located upstream of the *lsr* operon, and are transcribed divergently. The expression of *lsr* operon and its own *lsrRK* operon are inhibited by LsrR with directly binding to their promoters, while the effect of LsrR repression is released and the expression of *lsr* operon is activated when AI-2 is phosphorylated [17–19]. It has been well recognized that LsrR, as not only a direct regulator of the *lsr* operon, but a global effector of AI-2 QS system, regulates the expressions of hundreds of genes. However, the function of LsrR has been studied almost exclusively in model strains of *E. coli*. The influence of LsrR on gene expressions in MPEC and whether it is related to virulence regulation have been seldom reported.

In this study, to explore the function of LsrR in MPEC5, the transcriptional profile influenced by LsrR was analyzed using bioinformatics tools. The results showed the possible relationship between LsrR and biofilm formation. Biofilm assays verified that *lsrR* deletion significantly enhanced biofilm formation and EMSA assays further indicated that LsrR inhibits the biofilm formation ability of MPEC5 by directly binding to the promoter of *fimAICDFGH*. This study, for the first time, reports the role of LsrR in quorum sensing behaviors in MPEC, and might provide potential drug targets for the treatment and prevention of bovine mastitis.

## Results

***Transcriptomics profiling of lsrR-deficient mutant in E.coli MPEC5***.

**Fig. 1:**
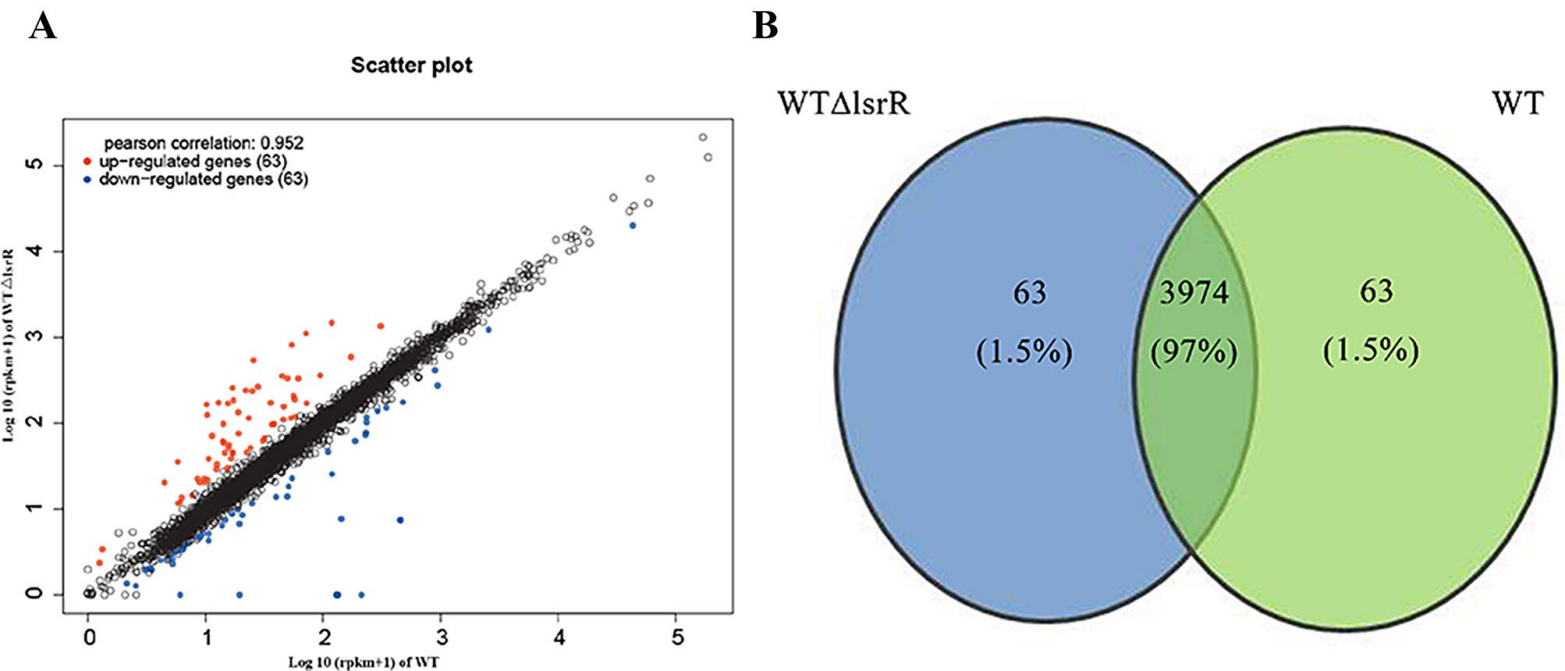
Identification and analysis of transcripts of WT strain MPEC5 and *lsrR*-knockout strain MPEC5Δ*lsrR*. (**A**) A scatter plot of DEGs expression between MPEC5 and MPEC5Δ*lsrR*. (**B**) A Venn plot of DEGs expression between MPEC5 and MPEC5Δ*lsrR*

To characterize the effect of LsrR on gene transcriptional profile in MPEC5, cDNA microarray experiments were carried out using the wild type and the *lsrR*-deletion strains. The transcriptomics sequencing of total RNA at exponential stage was analyzed and compared between the two strains (Fig. 1). The clean reads were compared with the reference genome, and genome comparison between WT strain and *lsrR*-deficient strain WTΔ*lsrR* was obtained with 94%～95% of mapping rate. A total of 126 differentially expressed genes (DEGs) in 4100 genes were identified, of which 63 were up-regulated (Table S1) and 63 were down-regulated (Table S2) in *lsrR*-deletion strains. Among these DEGs, several major represented pathways were associated with metabolism, genetic information processing, cell processes and environmental information processing. Among the virulence-related genes, the transcript levels of 7 Type I fimbriae encoding genes *fimAICDFGH*, which were co-transcribed, were increased significantly in *lsrR-deletion* strains (Table 1). In *E. coli*, Type 1 fimbriae are associated with the initial phase and maturation stage of biofilm formation, and we speculated that LsrR affects the biofilm formation of MPEC5 strain through the regulation of *fimAICDFGH* transcription.

**Table 1:** Mutation of *lsrR* gene led to up-regulation of transcription level of *fim* operon

Gene ID	Gene name	Products	Fold change
b4314	*fimA*	type I fimbriae major subunit	4.43
b4315	*fimI*	putative fimbriae protein FimI	1.97
b4316	*fimC*	type I fimbriae periplasmic chaperone	2.43
b4317	*fimD*	type I fimbriae usher protein	2.08
b4318	*fimF*	type I fimbriae minor subunit FimF	1.37
b4319	*fimG*	type I fimbriae minor subunit FimG	2.75
b4320	*fimH*	type I fimbriae D-mannose specific adhesin	2.43

### LsrR negatively regulates biofilm formation in strain MPEC5

**Fig. 2:**
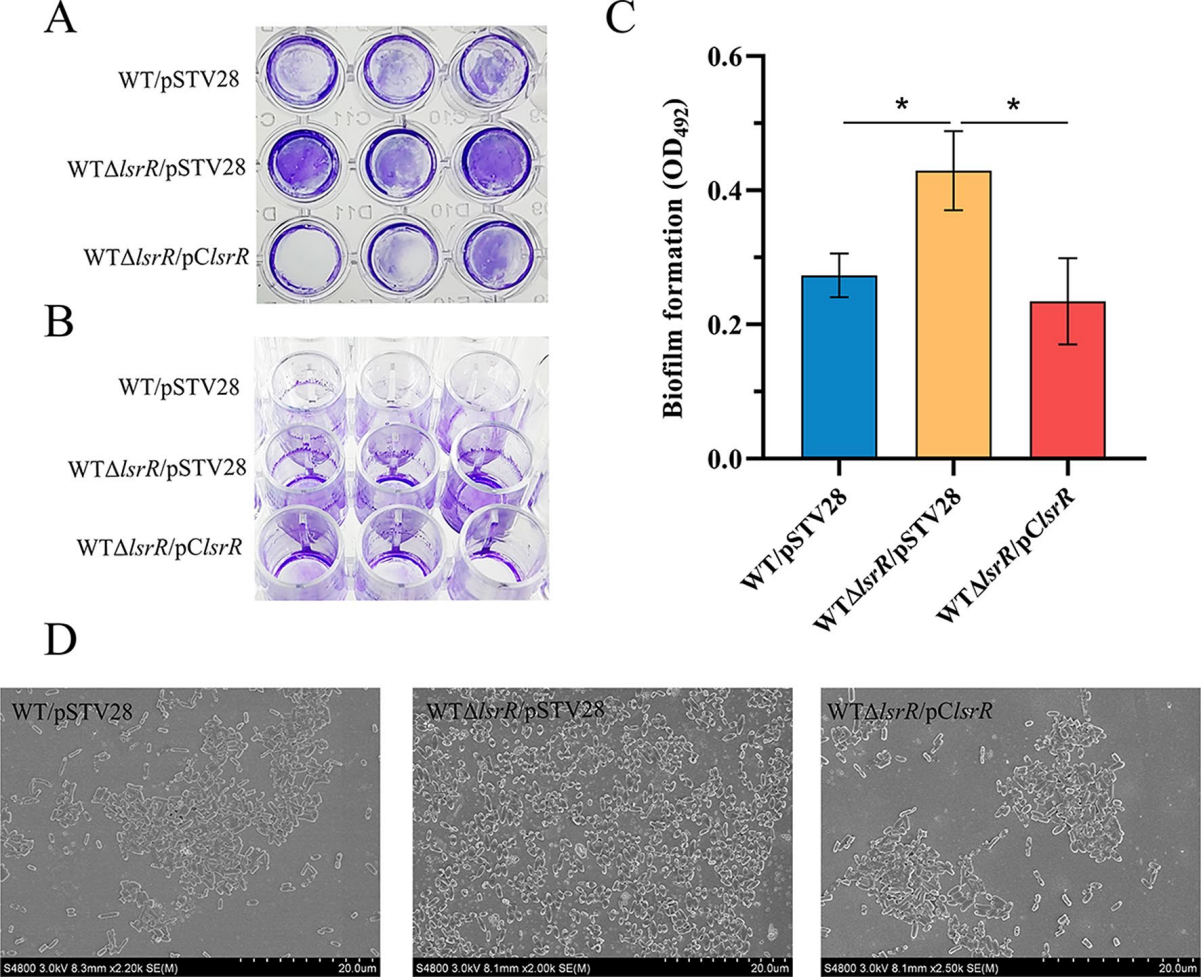
Detection of biofilm formation in strains WT/pSTV28, WTΔ*lsrR*/pSTV28 and WTΔ*lsrR*/pC*lsrR*. (**A**) A photograph of biofilm formation at the bottom of 96-well plates. (**B**) A photograph of biofilm formation in 96-well plate tube wall. (**C**) The quantitatively detection of the amount of biofilm formation by MicroELISA Autotrader. (**D**) A photograph of biofilm formation by SEM.* *P* < 0.05

To investigate whether LsrR has a regulatory effect on biofilm formation in strain MPEC5, the crystal violet staining assays of strains WT/pSTV28, WTΔ*lsrR*/pSTV28, and WTΔ*lsrR*/pC*lsrR* were performed. As shown in Fig. 2, biofilm formation on the bottom and lateral wall of 96-well plates was significantly increased in WTΔ*lsrR*/pSTV28 compared to WT/pSTV28, and the biofilms were restored in WTΔ*lsrR*/pClsrR. A MicroELISA Autoreader was used to further quantify the biofilm biomass (Fig. 2C), and the results showed that OD_492_ of strain WTΔ*lsrR*/pSTV28 (biofilm dissolved in 33% acetic acid solution) was significantly higher than that of the wild-type strain WT/pSTV28 and WTΔ*lsrR/*pC*lsrR*.

To further confirm the effect of LsrR on biofilm formation, a scanning electron microscope (SEM) assay was carried out among strains WT/pSTV28, WTΔ*lsrR*/pSTV28 and WTΔ*lsrR*/pClsrR (Fig. 2D). The biofilms of *l*srR-deficient mutant WTΔ*lsrR*/pSTV28 were plumpness and bacteria in biofilm gathered in piles. In contrast, the biofilm morphology of WT/pSTV28, and WTΔ*lsrR*/pClsrR were loose and adhesive, and the extracellular matters were less. These data suggest that LsrR negatively regulates biofilm formation in strain MPEC5.

### LsrR inhibits biofilm formation by decreasing transcription of the *fim* operon

**Fig. 3:**
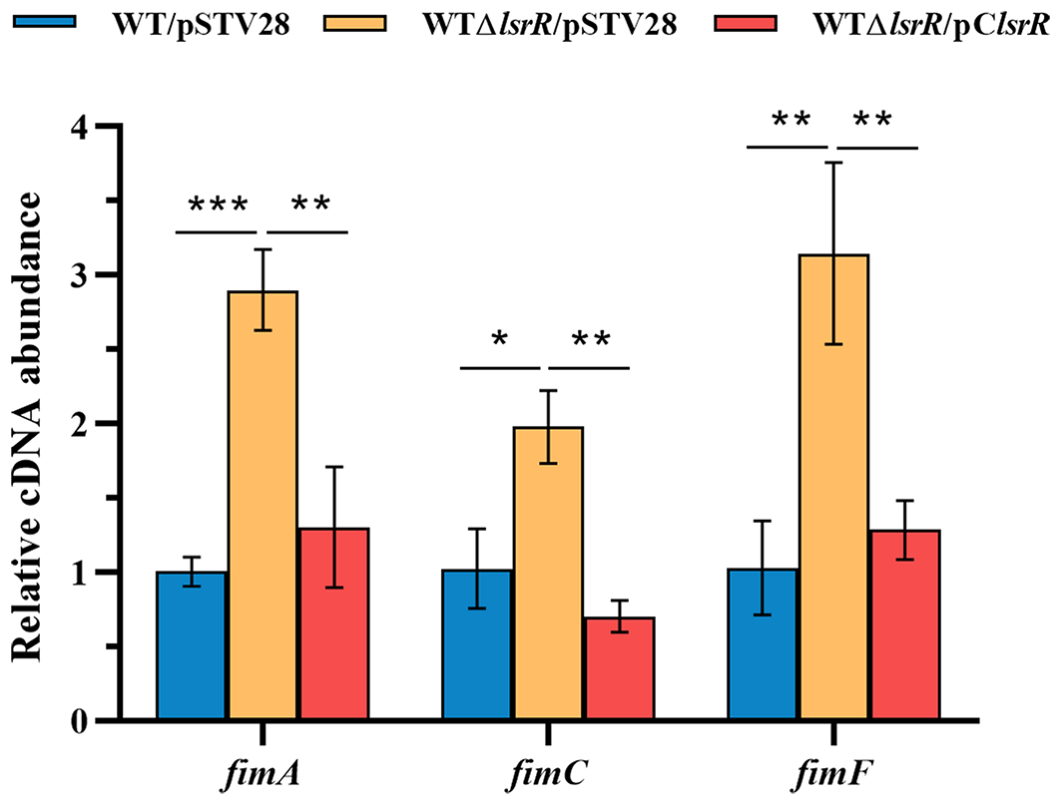
Transcription levels of biofilm-associated genes in strains WT/pSTV28, WTΔ*lsrR*/pSTV28 and WTΔ*lsrR*/pC*lsrR*. * *P* < 0.05, ** *P* < 0.01, *** *P* < 0.001

To explore how LsrR regulates biofilm formation in this MPEC strain, the transcript levels of several biofilm associated genes were measured by performing real-time RT-PCR assays. Previous studies showed that *lsrR* mutation significantly increased the expression of *wza*, which encodes a polysaccharide output protein [20]. Since the microarray data showed that there was no change in the transcriptions of other biofilm associated genes except the *fim* operon, the mRNA transcription levels of *fimA*, *fimC*, *fimF* and *wza* in the wild-type, *lsrR*-deletion and complementary strains were examined. As shown in Fig. 3, deletion of *lsrR* significantly increased transcript levels of *fimA*, *fimC*, and *fimF*, but had no obvious effect on the transcription of *wza* (data not shown). These results suggest that LsrR inhibits biofilm formation by down-regulating the transcription of *fimAICDFGH* operon in strain MPEC5.

### LsrR regulates transcriptional activity of *fim* operon by directly binding to the promoter region

**Fig. 4:**
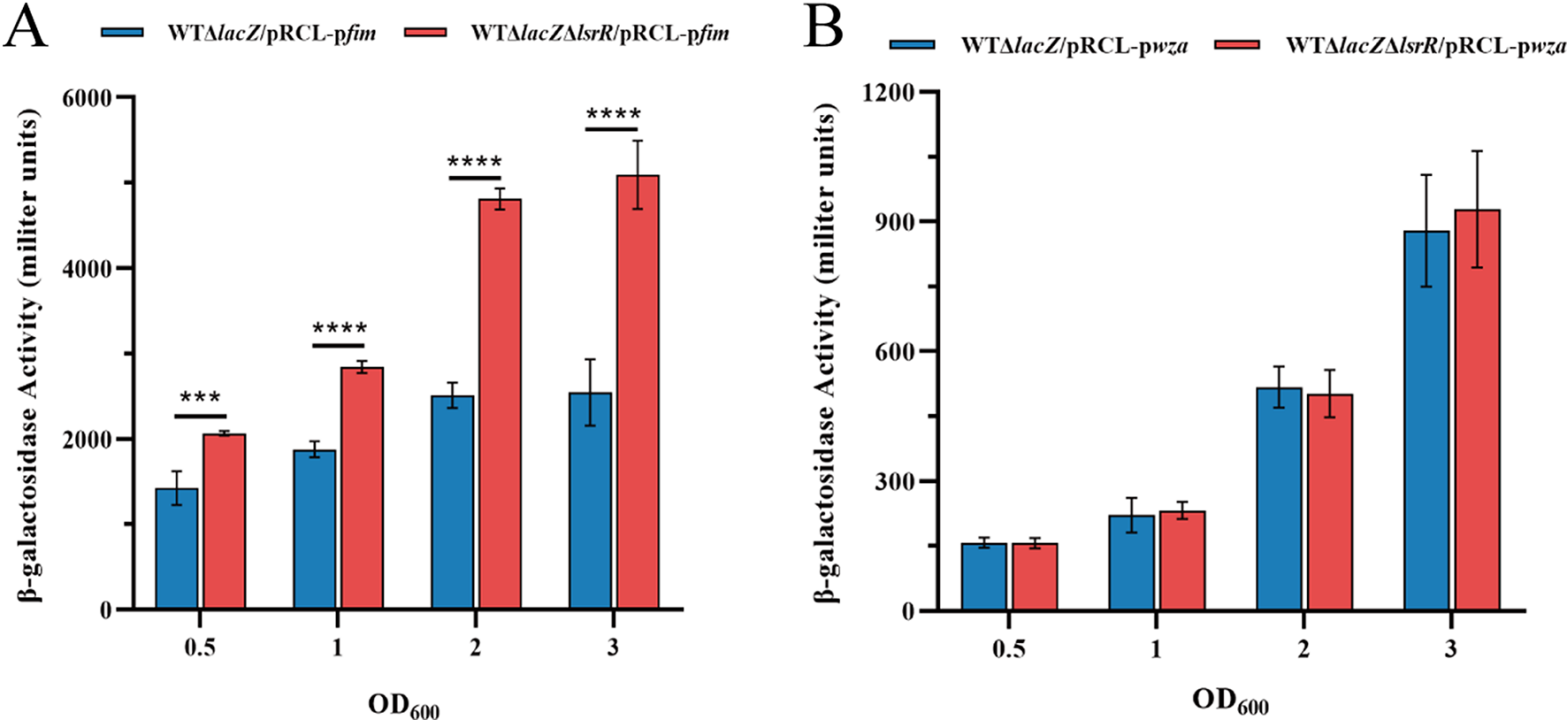
Determination of the transcription activities of *fim* and *wza* promoter. (**A**) β-galactosidase activity of WTΔ*lacZ*/pRCL-p*fim* and WTΔ*lsrR*Δ*lacZ*/pRCL-p*lsrR* strains. (**B**) β-galactosidase activity of WTΔ*lacZ*/pRCL-p*wza* and WTΔ*lsrR*Δ*lacZ*/pRCL-p*wza* strains. * *P* < 0.05

To further confirm the effect of *lsrR* mutation on transcription of *fim* operon and *wza*, β-galactosidase activity assays were performed to detected the transcriptional activities of *fimAICDFGH* and *wza* promoter during the whole growth cycle. As shown in Fig. 4. The expression level of *lacZ* in strain WTΔ*lsr*RΔ*lacZ*/pRCL-p*fim* was significantly higher than that of strain WTΔ*lacZ*/pRCL-p*fim* in exponential and stationary phase (Fig. 4A), indicating that LsrR inhibited transcription activity of the *fim* promoter. However, there was no significant difference in the transcriptional activity of *wza* promoter between WTΔ*lacZ*/pRCL-p*wza* and WTΔ*lacZ*Δ*lsr*R/pRCL-p*wza* strains during the whole growth cycle (Fig. 4B).

### LsrR bound to *fim AICDFGH promoters*

**Fig. 5:**
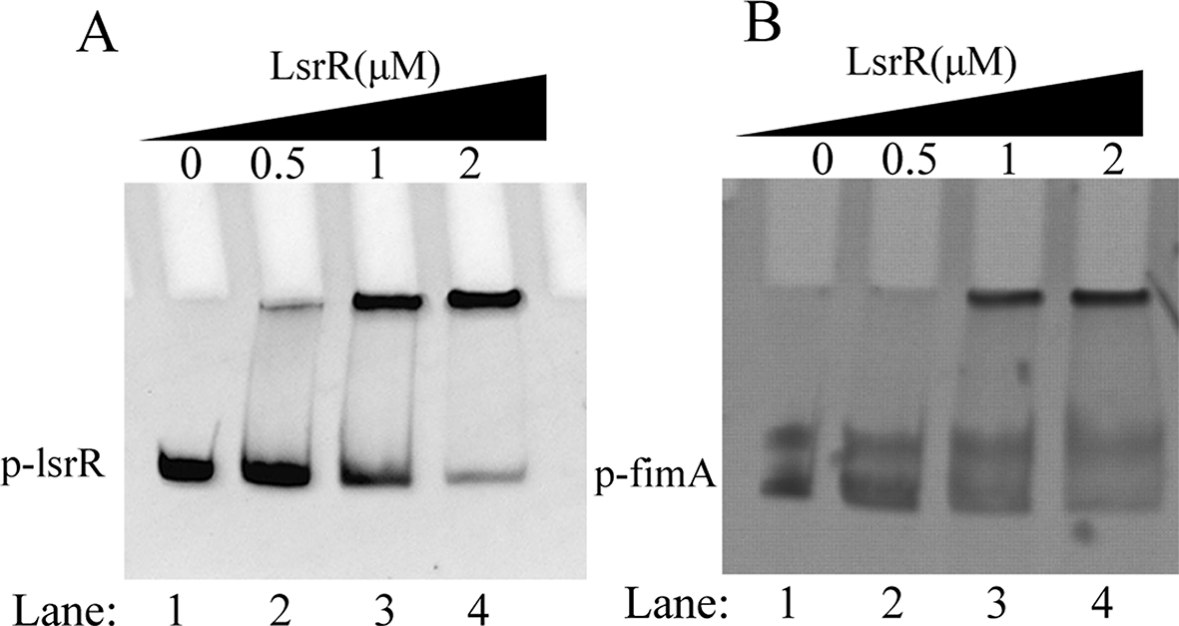
The binding ability of LsrR to the *fimA* promoter was determined by gel shift assays. Increasing LsrR amounts were incubated with probes *lsrR* and *fimA* promoters (p- lsrR and p- fimA). In each panel, from lanes (1) to (4), the LsrR concentrations were 0, 0.5, 1, and 2 µmol, respectively; the amounts of probes in all lanes were 100 ng. (**A**) Positive control group, the binding ability of LsrR and lsrR promoter; (**B**) The binding ability of LsrR and *fim* promoter

Our previous works have found that LsrR can directly bind to the promoter region containing high ratios of A and T of the *lsr* operon and several downstream target genes to modulate gene expression. A putative binding sequence of LsrR (5’-AACAATNN–NNAAAACTG-3’) was also found in the *fimAICDFGH* promoter region by sequence alignment. Therefore, EMSA were performed to verify whether LsrR directly binds to the promoter region of *fimAICDFGH*. As shown in Fig. 5, in the positive control group (Fig. 5A), the shifted band, which represented the complex formed by *plsrR* probes and LsrR protein, became more clearer as the LsrR protein concentration was increased, suggesting that LsrR protein blocked the migration of *lsrR* promoter in a dose-dependent manner. In consistent with the control group, the complex formed by *pfim* probes and LsrR protein, was enhanced with the increase of LsrR protein concentration (Fig. 5B), indicating that LsrR protein can also bind to the *pfim* promoter in a dose-dependent manner. These results confirmed that LsrR could negatively regulate the transcription of *fim* operon by directly binding to the promoter regions of *fimAICDFGH*, thus affecting type I fimbriae synthesis and the biofilm formation.

## Discussion

*E. coli* is a major cause of mastitis in cows, leading to acute or chronic intramammary infections and causing great economic losses to the dairy industry worldwide. In order to adapt to host environment, the pathogenic *E. coli* strain definitely undergoes alterations of the genome content and changes of virulence traits compared to the benign one. Although the pathogenic mechanisms of *E. coli* have been investigated thoroughly in previous studies, the gene regulation and virulence factors expression of MPEC associated with pathogenesis of mastitis are still largely unknown. Therefore, it is of great importance to investigate the gene regulation and virulence mechanisms in MPEC to prevent and cure bovine mastitis.

This study explored the regulatory role of LsrR in MPEC and demonstrated how LsrR affects virulence determinants including biofilm formation. The effect of LsrR in *E. coli* had only been reported by Li J et al. [21] in a model strain K12-W3110. In consistent with the previous work, our data also indicated that the expression of hundreds of genes was regulated by LsrR, further confirming the importance of LsrR as a global effector of QS system. The function of LsrR in bacterial biofilm formation has rarely been reported, and only been reported in a few bacteria except *E. coli* so far, including *Aggregatibacter actinomycetemcomitans* [22]. The biofilm formation capability of *lsrR* mutants in *A. actinomycetemcomitans* is significantly reduced compared to the wild type, but the molecular mechanism is still unclear. In this study, our data confirmed that deletion of *lsrR* resulted in a significant increase in biofilm formation. The biofilm formation of bacteria is a complex process that involves multiple regulatory systems and may vary between strains, which necessitates further research for verification. Our study provides new insight into the function and regulatory role of LsrR in *E. coli*. In particular, it is the first report of functional analysis of LsrR in MPEC strains and will help to find potential targets for the prevention and treatment of cow mastitis.

The biofilm formation in *E. coli* was affected by many factors, such as flagella, pili, polysaccharides, and adhesins [23]. Type 1 fimbriae, the most common and characteristic adhesion hormone in Enterobacteriaceae, regulates the host cell signaling pathway, bacterial infection, and biofilm formation [24]. Type 1 pili is necessary for adhesion to non-living surfaces in the initial stage of biofilm formation in *E. coli* [25, 26], and plays a crucial role in their pathogenicity. In this study, our data indicated that the transcriptions of 7 Type 1 fimbriae encoding genes were all significantly changed due to the inactivation of *lsrR*. In contrast, Li J et al. [21] showed that deletion of *lsrR* did not affect transcriptions of several known fimbria-related genes in the model *E. coli* strain K12-W3110, but the transcription of *wza*, encoding a polysaccharide output protein, was significantly up-regulated in *lsrR* or *lsrK* mutant. In addition, our results showed that significant differences in biofilm formation were observed in *lsrR* mutants both using crystal violet assay and SEM assays. However, the previous work by Li J et al. [21] only noticed the change of biofilm architecture by SEM. These results suggested that LsrR affected the biofilm formation by different regulatory pathways between the two *E. coli* strains and LsrR might have a stronger effect on biofilm formation capacity in strain MPEC5.

The mechanism of how LsrR regulates biofilm formation was investigated in this study. The results of transcriptomic sequencing showed that *lsrR* deletion significantly up-regulated the transcriptional levels of *fimAICDFGH*, but had no apparent change in transcription levels of other functional genes related to biofilm formation, indicating that LsrR regulates biofilm formation mainly by changing the expression of fimbria-related proteins. In addition, EMSA assays proved that LsrR negatively regulated the transcription levels of *fimAICDFGH* operon by directly binding to the promoter region of *fimAICDFGH.* The promoter region contains the putative LsrR -binding box consistent with our previous work. Although this study provided new evidence for the virulence regulation of LsrR in MPEC5, the downstream targets and the detailed regulatory mechanism of LsrR still need to be further explored in future.

## Conclusion

This study investigated the regulatory effect of LsrR in MPEC and first reported the molecular mechanism of LsrR regulating biofilm formation in mammary diseases caused by bacteria. The present findings provide direct evidence regarding the key role of LsrR in quorum sensing behavior of *E. coli*. These results provide important experimental basis and scientific research basis for preventing mastitis caused by *E. coli*.

## Materials and methods

### Bacterial strains, plasmids, and culture conditions

The bacterial strains and plasmids used in this study are listed in Table 2. The *E. coli* strain MPEC5 was obtained from a milk sample in dairy cows with clinical mastitis. The strains of wild type, mutant, and complement in this study are derived from our previous studies [5]. The *E. coli* strains were cultivated at 37 °C in Luria–Bertani (LB) broth (Oxoid, Basingstoke, UK) or on TSB agar plates containing 1.5% agar (Oxoid). When necessary, antibiotics (SangonBiotech, Shanghai, China) were added in to the media with the final concentrations for chloramphenicol at 15 µg/mL or kanamycin at 50 µg/mL.

**Table 2:** Strains and plasmids used in this study

Strain or plasmid	Relevant genotype	Reference or source
Strains		
*E. coli*		
DH5α	Clone host strain	Invitrogen
BL21 (DE3)	Strain of protein expression	Invitrogen
MPEC5	Wild type	[5]
WTΔ*lsrR*	MPEC5 *lsrR*-deletion mutant	[5]
WT/pSTV28	WT MPEC5 with the empty vector pSTV28, Cm^r^	[5]
WTΔ*lsrR*/pSTV28	WTΔ*lsrR* with the empty vector pSTV28, Cm^r^	[5]
WTΔ*lsrR*/pC*lsrR*	WTΔ*lsrR* with the complement plasmid *pClsrR*, Cm^r^	[5]
WTΔ*lacZ*	MPEC5 *lacZ*-deletion mutant	[5]
WTΔ*lsrR*Δ*lacZ*	MPEC5 *lacZ* and lsrR double deletion mutant	[5]
WTΔ*lacZ*/pRCL-p*fim*	WTΔ*lacZ* with plasmid pRCL- p*fim*, Cm^r^	This study
WTΔ*lsrR*Δ*lacZ*/pRCL-p*wza*	WTΔ*lsrR*Δ*lacZ* with plasmid pRCL -p*wza*, Cm^r^	This study
BL21 (DE3) /pET-*lsrR*	BL21 (DE3) with pET-*lsrR*, Kan^r^	This study
plasmids		
pRCL	promoterless *lacZ*, Cm^r^	[20]
pRCL-p*fim*	pRCL harboring *fim* promoter	This study
pRCL-p*wza*	pRCL harboring *wza* promoter	This study
pET-*lsrR*	pET28a (+) with *lsrR* gene, Kan^r^	[5]

Cm^r^, chloramphenicol-resistant; Kan^r^, kanamycin-resistant

## General DNA manipulation

Genomic DNA from *E. coli* MPEC5 was extracted by a standard protocol for Gram-negative bacteria. Plasmid DNA was obtained by employing a plasmid extraction kit (Promega, Madison, WI, USA), according to instructions or guidelines from the manufacturer. Taq or PrimeSTAR®Max DNA Polymerase (Takara Bio Inc., Dalian, China) was used in PCR amplification. A gel purification kit (Promega) was used to purify PCR products and DNA fragments according to instructions or guidelines from the manufacturer. DNA restriction enzyme (Takara, Dalian, Liaoning, China) digestion and T4 DNA ligase (Takara) ligation was performed by standard methods. Sequence analysis and primer design were carried out by Primer Premier 5.0 software, to predict the conserved domains of *lsrR* and to design the primers. The primers for amplifying nucleotide sequences in this study are listed in Table 3.

**Table 3:** Oligonucleotide primers used in this study

Primer name	Oligonucleotide (5′–3′)
rt-16s-f	TTTGAGTTCCCGGCC
rt-16s-r	CGGCCGCAAGGTTAA
rt-*fimA*-f	TCGCTGGCACAGGAAGGAG
rt-*fimA*-r	GTTTCTGAACTAAATGTCGCACC
rt-*fimC*-f	ATGCCGATGGTGTAAAGGA
rt-*fimC*-r	AATTGCGAGCTGTAGCGTAT
rt-*fimF*-f	CGGCGAAGCAATTTAACAA
rt-*fimF*-r	ACCCAACCTTTACGGCAGA
rt-*wza*-f	AAAACGGCGACCTCAACCA
rt-*wza*-r	TCTTCACTTCACCCATCACAAATAC
p*fim*-*Hind* III-f	CC*AAGCTT*TTGATTTAACTTATTGATAATA
p*fim*-*BamH* I-r	CG*GGATCC*CGCTGCTTTCCTTTCAAAAAACT
p*wza*-*Hind* III-f	CC*AAGCTT*AAAAGCCAGGGGCGGTAGCG
p*wza*-*BamH* I-r	CG*GGATCC*TGTTTATTTATCACTTTGGCAG
p-*lsrR*-f	ATTTCCCCCGTTCAGTTTTG
p-*lsrR*-r	AATTCATTCTTCACTTTGAA
p-*fim*-f	TTGATTTAACTTATTGATAATA
p-*fim*-r	GCTGCTTTCCTTTCAAAAAACT

f: is the forward primer; r: is the reverse primer, and the underlined base sequences is the recognition sites for restriction endonuclease

## Biofilm formation assays

The assays of biofilm formation were carried out based on the previous reports with some modifications [27, 28]. Briefly, the overnight cultures of WT/pSTV28, WTΔ*lsrR*/pSTV28, and WTΔ*lsrR*/pC*lsrR* were respectively diluted to 0.03 at the wavelength of 600 nm in 2 mL of fresh TSB broth, then incubated in 96 well-flat-bottom plates with 200 µL each well at 37℃ for 60 h without shaking. After discarding the planktonic cells and culture medium, the adherent bacteria at the bottom of the wells were cleaned with sterilized phosphate-buffered saline (PBS, pH 7.4) 3 times, and then dried naturally at room temperature for 10 h. The dried adherent bacteria were respectively treated as follows order: fixed with 100% methanol for 5 min, stained with 0.4% crystal violet for 18 min, washed with sterilized phosphate-buffered saline (PBS, pH 7.4) 3 times, and dissolved with 33% glacial acetic acid solution, then the absorbance at the wavelength of 492 nm was measured using a MicroELISA Autoreader (Thermo Scientific, Waltham, MA, USA). The test was repeated three times.

The test of scanning electron microscopy was carried out based on the previous reports with some modifications [29, 30]. For the assays of scanning electron microscopy (SEM XL20, Philips, Amsterdam, The Netherlands), the overnight cultures of WT/pSTV28, WTΔ*lsrR*/pSTV28, and WTΔ*lsrR*/pC*lsrR* were respectively diluted to 0.03 at the wavelength of 600 nm in fresh TSB. The diluted cultures, 5 mL per well, were transferred into a six-well-flat-bottom plate with a sterile coverslip at the bottom per well and cultured at 37℃ for 60 h without shaking. After cultivation, the biofilm bacteria on sterile coverslips from the bottom of the six-well-flat-bottom plate were washed three times with PBS solution and treated respectively through the following processing: fixed in 2.5% glutaraldehyde (Sangon, Shanghai, China) at 4 °C for 12 h, soaked in the PBS solution at room temperature for 20 min with two times, dehydrated with ethanol solution respectively for 20 min at 4 °C in order at different concentrations of 30%, 50%, 70%, 80%, 90%, and 100% (v/v). Then, biofilm bacteria on the coverslips were freeze-dried in carbon dioxide and the surface was sprayed with a gold film with approximately 10 nm thickness.

### RNA-seq, library generation, and transcriptome analysis

For the preparation of transcriptome sequencing samples, the overnight cultures of WT strain MPEC5 and mutation strain MPEC5Δ*lsrR* were respectively diluted to 0.03 at the wavelength of 600 nm in 4 mL fresh TSB, and grown in the culture tube until the exponential period (approximately 1 at the wavelength of 600 nm) at 37℃ with 220 rpm. Then the cells of WT strain MPEC5 and mutation strain MPEC5Δ*lsrR* were respectively collected by centrifugation and placed in dry ice and sent to Biozeron Biotechnology Co., Ltd. (Jiading, Shanghai, China) for transcriptome analysis and library construction.

RNA extraction and sequencing were carried out by Shanghai Ling En Biotechnology Co., LTD., and specific methods referred to a previous study [30]. Total RNA was extracted from the cells of MPEC5 and MPEC5Δ*lsrR* using TRIzol® Reagent according to instructions or guidelines from the manufacturer (Invitrogen, Carlsbad, CA, USA), and DNase I (TaKaRa, Beijing, China) was used to remove the genomic residual DNA. Then a 2100 Bioanalyzer (Agilent, Santa Clara, CA, USA) was used to determine the quality of obtained RNA and the ND-2000 (NanoDrop Technologies, Wilmington, USA) was used to quantify the amount of RNA. When the quality of RNA sample satisfied the following requirements: OD_260/280_ = 1.8 ~ 2.2, OD_260/230_ ≥ 2.0, RIN ≥ 6.5, 28 S:18 S ≥ 1.0, total mass > 10 µg, the sequencing libraries could be constructed using above high-quality RNA. TruSeq RNA sample preparation Kit from Illumina (San Diego, CA, USA) was used to prepare RNA-seq strand-specific libraries with 5 µg of total RNA. Briefly, after the rRNA was removed using Ribo-Zero rRNA removal kit (Epicenter, Madison, WI, USA), mRNA was fragmented using fragmentation buffer. According to the Illumina’s protocol, a series of steps were performed as follows in order: cDNA synthesis, end repair, A-base addition, and ligation of the Illumina-indexed adaptors. A library of the cDNA target fragments with the size of 200–300 bp was selected on 2% ultra-low range hyperagarose. Phusion DNA Polymerase (NEB, Ipswich, MA, USA) was used to amplified fragments by PCR for 15 PCR cycles. After being quantified by TBS380 (PicoGreen, Invitrogen, USA), the Illumina NovaSeq 6000 sequencing (150 bp × 2, Shanghai BIOZERON Co., Ltd) was used to sequence paired-end libraries.

For GO enrichment analysis, differentially expressed genes (DEGs) between WT and WTΔ*lsrR* strains [false discovery rate value < 0.05 and log_2_ (fold-change) > 1] were analyzed on the Gene Ontology Consortium website (geneontology.org). The Kyoto Encyclopedia of Genes and Genomes (KEGG) database (http://www.genome.jp/kegg) was used to analyze the KEGG pathway enrichment of DEGs with different pathways.

### RNA isolation, cDNA synthesis and quantitative real-time PCR analysis

Quantitative real-time PCR assays were carried out based on a previous study [5]. The cells of WT/pSTV28, WTΔ*lsrR*/pSTV28 and WTΔ*lsrR*/pC*lsrR* were collected and resuspended in RNase-free water containing 10 mg/mL lysozyme and 40 µg/mL lysostaphin (both from Sangon). After incubation at 37℃for 1 h, total RNA in the cells was extracted using Spin Column Bacteria Total RNA Purification Kit (Sangon). The EasyScript One-Step gDNA Removal and cDNA Synthesis SuperMix kit [TransGen Biotech (Beijing) Co. Ltd., Beijing, China] was used in reverse transcription assay. The TransStart Tip Green qPCR SuperMix kit (TransGen) was used in RT-qPCR assays.

### β-galactosidase assays

The strains of WTΔ*lacZ*/pRCL-p*fim*, WTΔ*lsrR*Δ*lacZ*/pRCL-p*lsrR*, WTΔ*lacZ*/pRCL-p*wza* and WTΔ*lsrR*Δ*lacZ*/pRCL-p*wza*, obtained from our previous study [5], were inoculated into 100 mL fresh TSB broth with 15 µg/mL chloramphenicol at the wavelength of 600 nm for the specified time. The cultivation cells were collected by a centrifugal method and resuspended in Z-buffer (Na_2_HPO_4_ •7 H_2_O at a final concentration of 16.1 g/L, NaH_2_PO_4_•H_2_O at a final concentration of 5.50 g/L, KCl at a final concentration of 0.75 g/L, and MgSO_4_•7 H_2_O at a final concentration of 0.246 g/L) to 1 mL and ortho-Nitrophenyl-β-galactoside(ONPG) was used as a substrate. The units of enzyme activity were expressed by the Miller Units, calculated through the following formula by Origin 8.0 software: [OD_420_ × 1000)/(OD_600_×Volume(mL)×Time (min)]. The experiment was repeated three times independently.

### Expression and purification of the LsrR protein

Isopropyl-β-D-thiogalactopyranoside (IPTG), final concentration of 0.5 mM, was added into the culture of BL21 (DE3)/pET-*lsrR* to induce expression of LsrR protein. LsrR fusion protein, carrying a 6-His tag, was purified by a HisTrap high-performance column referring to a previous study [5], and stored in 10% glycerol at -80℃ for electrophoretic mobility shift assay [30]. The purity of LsrR protein was detected by SDS-PAGE and its concentration was measured by an enhanced BCA protein assay kit (Beyotime, Shanghai, China).

### Electrophoretic mobility shift assay (EMSA)

The EMSAs were conducted as described previously [31]. The DNA promoter fragments of *fim*, *lsrR* gene were amplified from the WT genome of MPEC5 by probes primers p-*fim*-f/p-*fim-*r, p-*lsrR*-f/p-*lsrR-*r respectively, and incubated with various amounts of LsrR protein(0、0.5、1、2 µM) for 30 min at 25 °C in 4 µL 5×binding buffer (100 mM NaCl, 50 mM Tris–HCl, 3 mM magnesium acetate, 0.1 mM EDTA, 0.1 mM dithiothreitol, pH 7.5). After incubation, 2 µL 10×loading buffer with bromophenol blue was added into the mixture and then electrophoresed in a 4% native polyacrylamide gel in a 0.5×Tris–borate EDTA buffer. The band shifts were detected and analyzed on the basis of the manufacturer’s instructions in EMSA kit (Beyotime, Shanghai, China).

### Statistical analyses

Statistical analyses were conducted using GraphPad Prism 8.0 (GraphPad Software Inc., GraphPad Prism 8.0.1.244, San Diego, CA, USA). Statistically significant differences calculated by the unpaired two-tailed Student’s *t*-test are indicated: *, *P* < 0.05; **, *P* < 0.01; ***, *P* < 0.001; ****, *P* < 0.0001.

### Sequence data

Raw data of RNA-seq have been deposited into the NCBI Gene Expression database (https://www.ncbi.nlm.nih. gov) with the SRA accession number (PRJNA1062124).

### Electronic supplementary material

Below is the link to the electronic supplementary material.

Supplementary Material 1

Supplementary Material 2

## Data Availability

Data is provided within the manuscript or supplementary information files.

## Abbreviations

*E. coli*: *Escherichia coli*
MPEC: Mammary pathogenic *E. coli*
EPS: Extracellular polymers
QS: Quorum sensing
SEM: Scanning electron microscope
IPTG: Isopropyl β-D-thiogalactopyranoside
LB: Luria-Bertani
AI-2: Autoinducer 2
ONPG: Ortho-Nitrophenyl-β-galactoside
