# Supplementary material for: Role of LsrR in the regulation of biofilm formation in mammary pathogenic Escherichia coli
Source: BMC Vet Res. 2024 May 23;20:220. doi: 10.1186/s12917-024-04086-9 (PMC11112850; doi:10.1186/s12917-024-04086-9)
Supplement: Supplementary file 1 — Supplementary Material 1 [file 12917_2024_4086_MOESM1_ESM.docx]

Supplementary Materials for

**Role of LsrR in the regulation of biofilm formation in mammary pathogenic *Escherichia coli***

Li Xu ^1^, Wei Wang ^1^, Xin Zhang ^1^, Kai Ma ^1^, Hui Wang ^1,^ *, and Ting Xue ^1^^,^ *

^1^ School of Life Sciences, Anhui Agricultural University, Hefei, Anhui 230036, China

*Correspondence author: [wang28h@ustc.edu.cn](mailto:wang28h@ustc.edu.cn) and xuet@ahau.edu.cn.

**This PDF file includes:**

Tables S1

Tables S2

Figure S1

**Table S1.** Differentially expressed genes whose mRNA levels (log_2_FC) displayed > 1.0-fold increases in the *lsrR*-deficient mutant WTΔ*lsrR* compared with the WT strain.

| ID | Genes | Log_2_FC | Gene function |
| --- | --- | --- | --- |
| b1513 | *lsrA* | 4.46 | Autoinducer-2 ABC transporter ATP binding subunit |
| b1514 | *lsrC* | 4.16 | Autoinducer-2 ABC transporter membrane subunit LsrC |
| b2208 | *napF* | 4.01 | Ferredoxin-type protein |
| b1516 | *lsrB* | 3.98 | Autoinducer-2 ABC transporter periplasmic binding protein |
| b1517 | *lsrF* | 3.96 | 3-hydroxy-2,4-pentadione 5-phosphate thiolase |
| b1515 | *lsrD* | 3.86 | Autoinducer-2 ABC transporter membrane subunit LsrD |
| b2207 | *napD* | 3.74 | NapA signal peptide-binding chaperone NapD |
| b1518 | *lsrG* | 3.65 | (4S)-4-hydroxy-5-phosphonooxypentane-2,3-dione isomerase |
| b1227 | *narI* | 3.54 | Nitrate reductase A subunit gamma |
| b1226 | *narJ* | 3.52 | Nitrate reductase 1 molybdenum cofactor assembly chaperone |
| b2206 | *napA* | 3.50 | Periplasmic nitrate reductase subunit NapA |
| b2203 | *napB* | 3.31 | Periplasmic nitrate reductase cytochrome c550 protein |
| b1511 | *lsrK* | 3.30 | Autoinducer-2 kinase |
| b4070 | *nrfA* | 3.01 | Cytochrome c552 nitrite reductase |
| b1225 | *narH* | 2.89 | Nitrate reductase A subunit beta |
| b2205 | *napG* | 2.89 | Ferredoxin-type protein NapG |
| b1705 | *ydiE* | 2.85 | PF10636 family protein YdiE |
| b4813 | *_-* | 2.84 | - |
| b1512 | *lsrR* | 2.77 | DNA-binding transcriptional repressor LsrR |
| b1223 | *narK* | 2.75 | Nitrate:nitrite antiporter NarK |
| b3366 | *nirD* | 2.48 | Nitrite reductase (NADH) small subunit |
| b2202 | *napC* | 2.45 | Periplasmic nitrate reductase cytochrome c protein |
| b3808 | *yzcX* | 2.37 | Protein YzcX |
| b2204 | *napH* | 2.35 | Ferredoxin-type protein NapH |
| b2201 | *ccmA* | 2.31 | Fytochrome c maturation protein A |
| b2199 | *ccmC* | 2.22 | Cytochrome c maturation protein C |
| b4314 | *fimA* | 2.14 | Type 1 fimbriae major subunit |
| b2200 | *ccmB* | 2.12 | Cytochrome c maturation protein B |
| b1224 | *narG* | 2.05 | Nitrate reductase A subunit alpha |
| b3365 | *nirB* | 1.96 | Nitrite reductase (NADH) large subunit |
| b0782 | *moaB* | 1.95 | Protein MoaB |
| b0783 | *moaC* | 1.91 | Cyclic pyranopterin monophosphate synthase |
| b2198 | *ccmD* | 1.90 | Cytochrome c maturation protein D |
| b1221 | *narL* | 1.79 | DNA-binding transcriptional dual regulator NarL |
| b2197 | *ccmE* | 1.78 | Periplasmic heme chaperone |
| b4071 | *nrfB* | 1.78 | Periplasmic nitrite reductase penta-heme c-type cytochrome |
| b0784 | *moaD* | 1.74 | Molybdopterin synthase sulfur carrier subunit |
| b2196 | *ccmF* | 1.69 | Holocytochrome c synthase CcmF component |
| b4073 | *nrfD* | 1.49 | Putative menaquinol-cytochrome c reductase subunit NrfD |
| b4072 | *nrfC* | 1.48 | Putative menaquinol-cytochrome c reductase 4Fe-4S subunit |
| b4319 | *fimG* | 1.47 | Type 1 fimbriae minor subunit FimG |
| b2195 | *ccmG* | 1.45 | Disulfide oxidoreductase CcmG |
| b3908 | *sodA* | 1.42 | Superoxide dismutase (Mn) |
| b3408 | *feoA* | 1.38 | Ferrous iron transport protein A |
| b3479 | *nikD* | 1.31 | Ni(2(+)) ABC transporter ATP binding subunit NikD |
| b2424 | *cysU* | 1.30 | Sulfate ABC transporter inner membrane subunit CysU |
| b3478 | *nikC* | 1.30 | Ni(2(+)) ABC transporter membrane subunit NikC |
| b4316 | *fimC* | 1.28 | Type 1 fimbriae periplasmic chaperone |
| b4320 | *fimH* | 1.28 | Type 1 fimbriae D-mannose specific adhesin |
| b0785 | *moaE* | 1.26 | Molybdopterin synthase catalytic subunit |
| b2422 | *cysA* | 1.25 | Sulfate/thiosulfate ABC transporter ATP binding subunit |
| b3477 | *nikB* | 1.15 | Ni(2(+)) ABC transporter membrane subunit NikB |
| b2423 | *cysW* | 1.14 | Sulfate ABC transporter inner membrane subunit CysW |
| b2764 | *cysJ* | 1.14 | Sulfite reductase, flavoprotein subunit |
| b2425 | *cysP* | 1.13 | Thiosulfate ABC transporter periplasmic binding protein CysP |
| b0781 | *moaA* | 1.13 | GTP 3',8'-cyclase |
| b4437 | *_-* | 1.11 | - |
| b4721 | *ytiD* | 1.10 | Protein YtiD |
| b4317 | *fimD* | 1.06 | Type I fimbriae usher protein |
| b4720 | *ytiC* | 1.05 | Protein YtiC |
| b1222 | *narX* | 1.05 | Sensor histidine kinase NarX |
| b3410 | *feoC* | 1.01 | Ferrous iron transport protein FeoC |
| b2751 | *cysN* | 1.01 | Sulfate adenylyltransferase subunit 1 |

**Table S2**. Differentially expressed genes whose mRNA levels (log_2_FC) displayed > 1.0-fold decreases in the *lsrR*-deficient mutant WTΔ*lsrR* compared with the WT strain.

| ID | Genens | Log_2_FC | Gene function | |  |
| --- | --- | --- | --- | --- | --- |
| b1994 | *insH6* | -17.69 | | IS5 family transposase and trans-activator | |
| b2982 | *insH9* | -17.02 | | IS5 family transposase and trans-activator | |
| b0259 | *insH1* | -17.00 | | IS5 family transposase and trans-activator | |
| b2030 | *insH7* | -17.00 | | IS5 family transposase and trans-activator | |
| b2192 | *insH8* | -17.00 | | IS5 family transposase and trans-activator | |
| b0552 | *insH2* | -17.00 | | IS5 family transposase and trans-activator | |
| b1331 | *insH4* | -17.00 | | IS5 family transposase and trans-activator | |
| b3505 | *insH11* | -17.00 | | IS5 family transposase and trans-activator | |
| b0656 | *insH3* | -17.00 | | IS5 family transposase and trans-activator | |
| b4711 | *insH21* | -17.00 | | IS5 family transposase and trans-activator | |
| b3218 | *insH10* | -17.00 | | IS5 family transposase and trans-activator | |
| b4294 | *insA7* | -14.18 | | IS1 family repressor protein InsA | |
| b1370 | *insH5* | -12.32 | | IS5 family transposase and trans-activator | |
| b0275 | *insA3* | -6.15 | | IS1 family protein InsA | |
| b0265 | *insA2* | -6.13 | | IS1 family protein InsA | |
| b4516 | *insA4* | -4.42 | | IS1 family protein InsA | |
| b0012 | *mbiA* | -2.53 | | Uncharacterized protein MbiA | |
| b0988 | *insB4* | -2.27 | | IS1 family protein InsB | |
| b0334 | *prpD* | -1.89 | | 2-methylcitrate dehydratase | |
| b4532 | *hicA* | -1.77 | | MRNA interferase toxin HicA | |
| b3104 | *yhaI* | -1.73 | | Putative inner membrane protein | |
| b2975 | *glcA* | -1.70 | | Glycolate/lactate:H(+) symporter GlcA | |
| b4253 | *yjgL* | -1.67 | | Protein YjgL | |
| b0274 | *insB3* | -1.67 | | IS1 family protein InsB | |
| b0333 | *prpC* | -1.60 | | 2-methylcitrate synthase | |
| b1438 | *hicB* | -1.60 | | DNA-binding transcriptional repressor HicB | |
| b0264 | *insB2* | -1.58 | | IS1 family protein InsB | |
| b2838 | *lysA* | -1.53 | | Diaminopimelate decarboxylase | |
| b4608 | - | -1.53 | | - | |
| b2977 | *glcG* | -1.45 | | Putative heme-binding protein GlcG | |
| b3900 | *frvA* | -1.40 | | Putative PTS enzyme IIA component FrvA | |
| b0331 | *prpB* | -1.39 | | 2-methylisocitrate lyase | |
| b0531 | *sfmC* | -1.38 | | Putative fimbrial chaperone SfmC | |
| b1408 | *ynbA* | -1.36 | | Alcohol phosphatidyl transferase domain protein YnbA | |
| b3025 | *qseB* | -1.30 | | DNA-binding transcriptional activator QseB | |
| b4330 | *yjiH* | -1.26 | | Uncharacterized protein YjiH | |
| b3369 | *yhfL* | -1.23 | | DUF4223 domain-containing lipoprotein YhfL | |
| b2061 | *wzb* | -1.23 | | Protein-tyrosine phosphatase | |
| b0248 | *yafX* | -1.22 | | Protein YafX | |
| b1500 | *safA* | -1.21 | | Two-component system connector SafA | |
| b1529 | *marC* | -1.20 | | Inner membrane protein MarC | |
| b2976 | *glcB* | -1.20 | | Malate synthase G | |
| b0256 | *insI1* | -1.18 | | IS30 family transposase | |
| b0335 | *prpE* | -1.17 | | Propionyl-CoA synthetase | |
| b3579 | *yiaO* | -1.14 | | Na(+) symporter - periplasmic binding protein | |
| b0319 | *yahE* | -1.13 | | DUF2877 domain-containing protein YahE | |
| b3587 | *yiaW* | -1.13 | | DUF3302 domain-containing protein YiaW | |
| b4682 | *yqcG* | -1.12 | | Cell envelope stress response protein YqcG | |
| b1014 | *putA* | -1.11 | | 1-pyrroline-5-carboxylate dehydrogenase PutA | |
| b0718 | *ybgQ* | -1.11 | | Putative fimbrial usher protein YbgQ | |
| b4457 | - | -1.10 | | - | |
| b4468 | *glcE* | -1.09 | | Glycolate dehydrogenase, putative FAD-binding subunit | |
| b1423 | *ydcJ* | -1.07 | | DUF1338 domain-containing protein YdcJ | |
| b4097 | *phnK* | -1.06 | | Carbon-phosphorus lyase subunit PhnK | |
| b1550 | *gnsB* | -1.05 | | Protein GnsB | |
| b3119 | *tdcR* | -1.05 | | DNA-binding transcriptional activator TdcR | |
| b2058 | *wcaB* | -1.04 | | Colanic acid biosynthesis acetyltransferase WcaB | |
| b0368 | *tauD* | -1.03 | | Taurine dioxygenase | |
| b1058 | *yceO* | -1.03 | | DUF2770 domain-containing protein YceO | |
| b1984 | - | -1.03 | | - | |
| b4099 | *phnI* | -1.03 | | Carbon-phosphorus lyase core complex subunit PhnI | |
| b1802 | *yeaW* | -1.01 | | Carnitine monooxygenase subunit YeaW | |
| b0466 | *ybaM* | -1.01 | | DUF2496 domain-containing protein YbaM | |


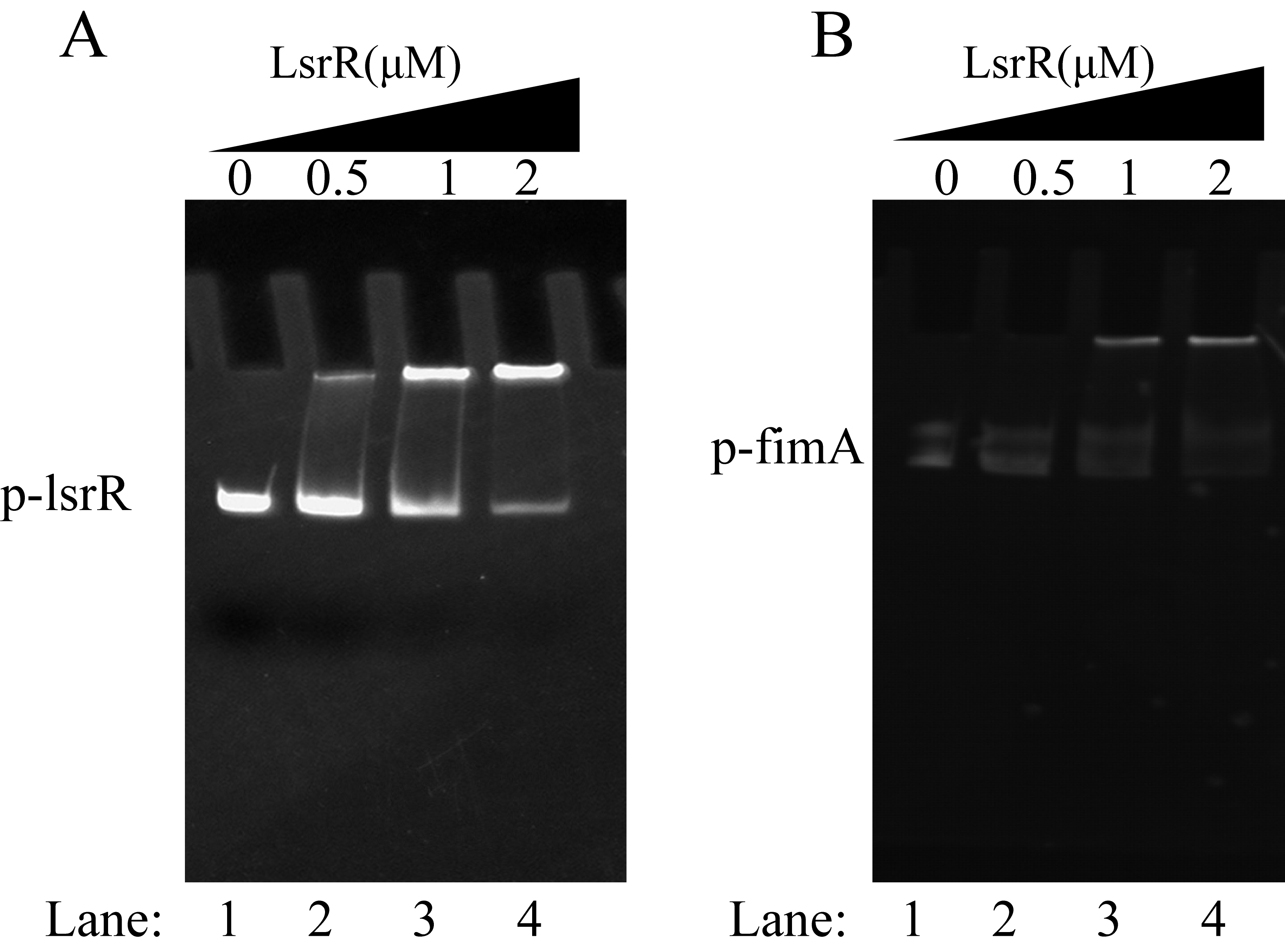


Fig. S1. The binding ability of LsrR to the *fimA* promoter was determined by gel shift assays. Increasing LsrR amounts were incubated with probes *lsrR* and *fimA* promoters (p- *lsrR* and p- *fimA*). In each panel, from lanes (1) to (4), the LsrR concentrations were 0, 0.5, 1, and 2 μmol, respectively; the amounts of probes in all lanes were 100 ng. (A) Positive control group, the binding ability of LsrR and *lsrR* promoter; (B) The binding ability of LsrR and *fim* promoter.
